# Computational protein design repurposed to explore enzyme vitality and help predict antibiotic resistance

**DOI:** 10.3389/fmolb.2022.905588

**Published:** 2023-01-09

**Authors:** Eleni Michael, Rémy Saint-Jalme, David Mignon, Thomas Simonson

**Affiliations:** Laboratoire de Biologie Structurale de la Cellule (CNRS UMR7654), Ecole Polytechnique, Palaiseau, France

**Keywords:** Proteus software, dihydrofolate reductase, molecular mechanics, Monte Carlo, adaptive landscape flattening

## Abstract

In response to antibiotics that inhibit a bacterial enzyme, resistance mutations inevitably arise. Predicting them ahead of time would aid target selection and drug design. The simplest resistance mechanism would be to reduce antibiotic binding without sacrificing too much substrate binding. The property that reflects this is the enzyme “vitality”, defined here as the difference between the inhibitor and substrate binding free energies. To predict such mutations, we borrow methodology from computational protein design. We use a Monte Carlo exploration of mutation space and vitality changes, allowing us to rank thousands of mutations and identify ones that might provide resistance through the simple mechanism considered. As an illustration, we chose dihydrofolate reductase, an essential enzyme targeted by several antibiotics. We simulated its complexes with the inhibitor trimethoprim and the substrate dihydrofolate. 20 active site positions were mutated, or “redesigned” individually, then in pairs or quartets. We computed the resulting binding free energy and vitality changes. Out of seven known resistance mutations involving active site positions, five were correctly recovered. Ten positions exhibited mutations with significant predicted vitality gains. Direct couplings between designed positions were predicted to be small, which reduces the combinatorial complexity of the mutation space to be explored. It also suggests that over the course of evolution, resistance mutations involving several positions do not need the underlying point mutations to arise all at once: they can appear and become fixed one after the other.

## 1 Introduction

When bacteria are challenged with an antibiotic that inhibits an essential enzyme, mutations appear that reduce antibiotic effectiveness (Condra et al., 1995; Podnecky et al., 2017; Thompson et al., 2020). Understanding and predicting them would aid in target selection and drug design. Resistance to an enzyme inhibitor can involve several mechanisms. Perhaps the simplest would be to reduce the antibiotic binding without sacrificing too much substrate binding. The property that reflects this is the enzyme “vitality”, defined here as the difference between the inhibitor and substrate binding free energies. To predict potential resistance mutations that use this mechanism, we propose methodology borrowed from computational protein design (CPD), a powerful tool to explore and characterize large sets of enzyme mutations (Stoddard, 2016; Leman et al., 2020; Michael and Simonson, 2022). The method uses a Monte Carlo (MC) exploration of mutation space and gives estimates of ligand binding, thanks to an adaptive flattening of a free energy landscape (Villa et al., 2018). This allows us to rank mutations according to enzyme vitality and predict ones that might provide resistance through the simple mechanism considered here. The simulation method was recently successful for a related problem, allowing an accurate prediction of ligand binding to redesigned variants of an aminoacyl-tRNA synthetase (Opuu et al., 2020).

As an illustration, we considered dihydrofolate reductase (DHFR), an essential enzyme targeted by several antibiotics (Thompson et al., 2020). DHFR generates tetrahydrofolate by transfering a hydride from NADPH to dihydrofolate (DHF) (Stryer, 1988; Adamczyk et al., 2011). Tetrahydrofolate derivatives are then consumed as one-carbon unit donors in a variety of biosyntheses, including those of thymidine and DNA. DHFR is targeted by both antibacterial and anticancer drugs. We considered *Escherichia coli* DHFR and its binding of trimethoprim (TMP), a competitive inhibitor commonly used as an antibiotic (Bugrysheva et al., 2017). Using MC simulations and adaptive landscape flattening, we mapped out enzyme vitality changes over sequence space.

We applied an established CPD model, where the protein and ligand were described by molecular mechanics and solvent was treated as a dielectric continuum (Mignon et al., 2020). We developed force field parameters for DHF and TMP, which are of general interest. We enumerated allowed conformers, or “rotamers” of each ligand within the binding pocket. We then used the model to explore DHFR vitality. We used long MC simulations to sample mutations (and conformations) of the 20 residues closest to the substrate position. Each residue was first mutated separately, with the others keeping their native types. Ten amino acid positions led to significant vitality gains, of around 1–4 kcal/mol. Then, 51 pairs of positions were mutated, covering all pairs of active site positions close enough to interact directly with each other. We also explored one quartet of positions, as a larger-scale illustration. Out of 104976 possible quartet sequences, 80083 (76%) were extensively sampled over the course of the MC simulation. Although our study is predictive and meant to illustrate the methodology, comparison to known resistance mutants provides some validation. Of seven resistance mutations that involve active site positions and are experimentally known, five were correctly recovered, with significant vitality gains.

By comparing the single-position and pair results, we also determined the direct coupling strengths between positions. Coupling can lead to correlated mutations during evolution and might facilitate bacterial resistance (Cocco et al., 2018; Allen and Waclaw, 2019). Most were very small, below .25 kcal/mol. Only for a few pairs in close proximity, couplings as large as 2–3 kcal/mol were predicted for the largest side chain types. On a practical side, weak direct couplings mean that the MC exploration can safely be done a few positions at a time (as here), instead of trying to sample 20 positions all at once in a single simulation, a huge combinatorial problem. On a fundamental side, the weak couplings suggest that over the course of evolution, when resistance mutations involve several positions, the underlying point mutations do not need to arise all at once—an improbable event. Rather, they can appear and become fixed in the population one after the other.

## 2 Methods

### 2.1 Enzyme vitality with adaptive landscape flattening

Enzyme vitality is defined by the competition between antibiotic and binding of the substrate (or the transition state) (Ishikita and Warshel, 2008; Singh et al., 2012; Jindal et al., 2017). Here, we consider the antibiotic TMP and the substrate DHF. The corresponding affinities of a variant, relative to the native DHFR, are denoted $\Delta G_{\text{Bind}}^{\text{TMP}}$ and $\Delta G_{\text{Bind}}^{\text{DHF}}$ , respectively. The vitality (relative to native) is defined here as

$$\Delta G_{\text{Vit}}=\Delta G_{\text{Bind}}^{\text{DHF}}-\Delta G_{\text{Bind}}^{\text{TMP}}-{\left(\Delta G_{\text{Bind}}^{\text{DHF}}-\Delta G_{\text{Bind}}^{\text{TMP}}\right)}_{\text{Native}}$$ (1)

The subscript Native on the rightmost parenthesis indicates that the affinities for native DHFR are subtracted out. With this definition, the native sequence has a vitality of zero and the best vitalities are large and negative.

To obtain the relative affinities in Eq. 1, we flatten the free energy landscape of three systems: apo DHFR and its complexes with DHF and TMP. All three systems include the NADPH cofactor. During an MC simulation of each system, a bias potential *E* ^bias^ is gradually constructed that depends on the side chain type at each mutating position (Villa et al., 2018). The form of the bias is given further on. Eventually, all types appear with comparable probabilities, and therefore the energy landscape has been flattened. In the case of perfect flattening, all types have exactly equal probabilities, and the bias potential of each sequence is equal to its relative folding free energy Δ*G*_Fold_, up to a sign change and a constant:

$$\Delta G_{\text{Fold }}=-\Delta E^{\text{Bias}}+constant$$ (2)

The bias is then included in a second simulation, from which we obtain the “biased” probabilities *p*(*S*) of each sequence variant *S*. Finally, the unbiased free energy of each sequence is obtained by subtracting out the bias:

$$\Delta G\left(S\right)=-k_{B}T\ln\frac{p\left(S\right)}{p\left(S_{\text{ref}}\right)}-\Delta E^{\text{Bias}}\left(S\right),$$ (3)

where *S*_ref_ is a designated reference sequence, such as the native sequence. From the relative free energies, we can estimate relative affinities by subtracting apo and holo results, and relative vitalities by subtracting DHF and TMP results.

### 2.2 Ligand tautomers and protonation states

**FIGURE 1:**
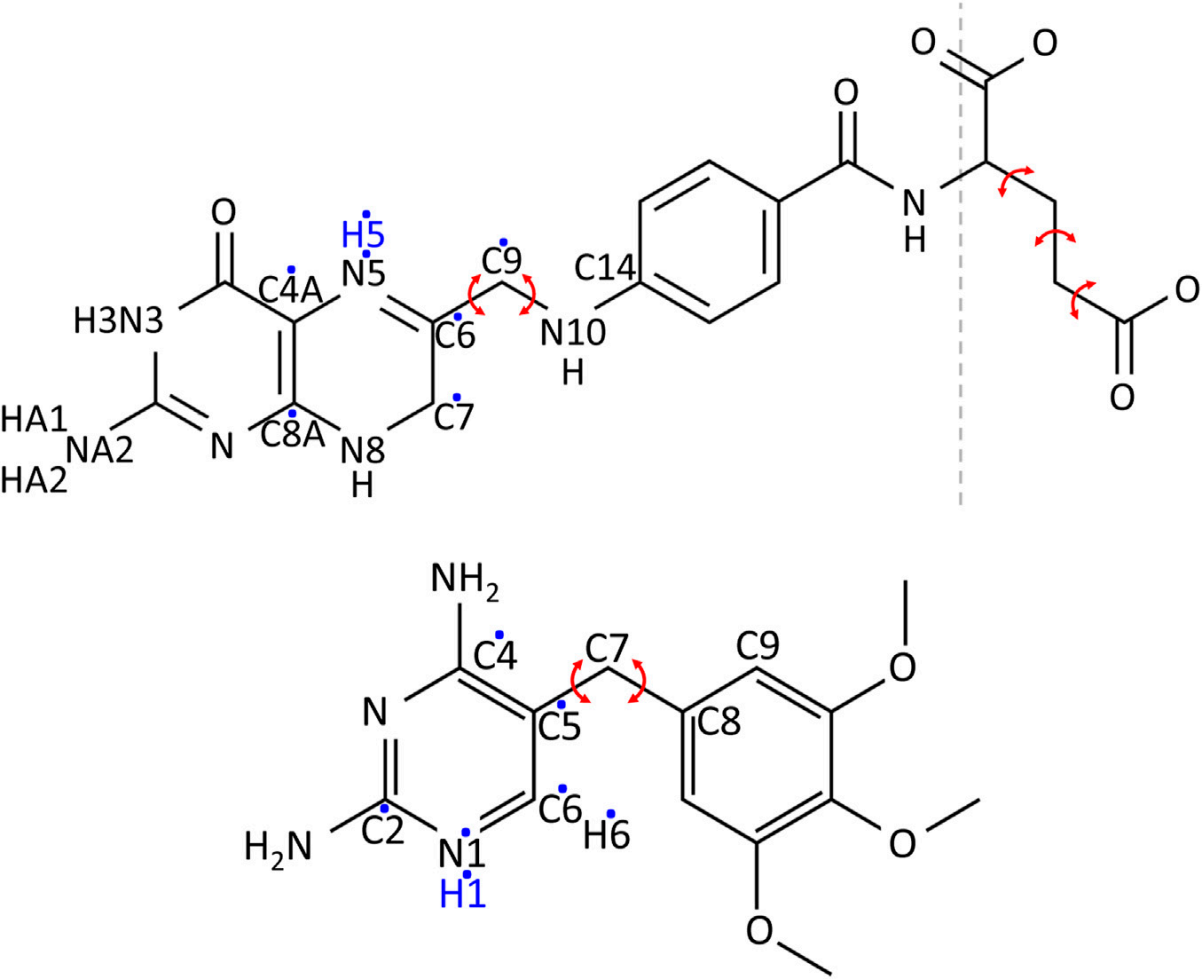
Chemical structures of DHF (above) and TMP (below). Red arrows indicate the bond rotations defining ligand conformers. A dashed line separates DHF into the fragment used in Gaussian calculations (left) and the glutamic acid fragment (right). Hydrogens added in the protonated form of the ligands are in blue. Dots indicate the atoms among which the extra charge is distributed upon protonation.

The determination of the protonation states and tautomeric forms of DHF, TMP, and NADPH was done by considering the known properties of analogous molecules and by using information from sequence alignments, 3D structure inspection, and statistical analysis of interactions during MD simulations of protein-ligand complexes. In particular, a high-resolution neutron structure solved at neutral pH is available (PDB code 4PDJ) (Wan et al., 2014), where many hydrogen atoms can be seen. Atom names for DHF and TMP are shown in Figure 1. For DHF, the N3-protonated tautomer is clearly seen and was adopted here. For N5, the pK_*a*_ is known to be 6.5 (Wan et al., 2014). We adopted the N5-protonated form here, because it is considered an important intermediate along the reaction pathway, prior to hydride transfer from NADPH (Wan et al., 2014). For TMP, we selected the N1-protonated form, which has a total charge of +1, because a close interaction with the conserved, negative residue Asp27 is seen in several crystal structures. For NADPH, we selected the form with its terminal phosphate deprotonated. This form appears clearly in the neutron structure. In addition, we surveyed 61 experimental structures of NADPH in complex with DHFR, and did a statistical analysis of the phosphate environment. We also performed MD simulations with the phosphate either singly-protonated or fully-deprotonated and compared the phosphate interactions in each case to the PDB survey. This analysis also strongly supports the fully-deprotonated phosphate model. Details are in Supplementary Table S1.

### 2.3 Ligand force field

Atomic charges of the ligands were obtained using *ab initio* calculations with Gaussian 9.0 (Frisch et al., 2009). Calculations were done for the entire TMP and for a DHF moiety that excludes the glutamic acid portion. Input coordinates were taken from crystal structures: PDB code 1RX2 (Sawaya and Kraut, 1997) for DHF and 6XG5 (Manna et al., 2021) for TMP. We did energy minimization, then extracted charges with electrostatic potential fitting (Cornell et al., 1995). We used the HF//6-31G* method for neutral forms of each molecule and HF//6-31G** for cationic forms. For each cationic form, we compared the charges with the corresponding, deprotonated, neutral form and identified the atoms with the greatest charge modifications. The new, cationic charges were applied only to the most affected atoms (7 atoms in all cases), while the other charges kept their neutral values. The small excess charge thus created was eliminated by adding a uniform increment to the same seven atoms, so that the total charge was +1.

Equilibrium geometry parameters were taken from the Gaussian-minimized geometries, or by analogy with standard groups (Cornell et al., 1995). Atom types and stiffness constants were determined by analogy with standard groups. One group with less obvious analogs was the atom C7 and its neighbors in the pteridine portion of DHF. The possibility of ring puckering at this atom was considered. In *ab initio* structures minimized in vacuum, C7 deviated from the plane of the ring by about .5 Å, with a dihedral angle for atoms C6-C7-N8-C8A equal to −28.0°. The energy needed to flatten the ring was between .5 and 2.4 kcal/mol, depending on the quantum mechanical method and basis set. In contrast, a survey of 30 experimental structures of *E. Coli* DHFR in complex with DHF gave a mean absolute dihedral angle of 1.3 ± 1.0° and a mean C7 distance from the ring plane of just .04 ± .03 Å. Details are in Supplementary Figure S1. Finally, a quantum calculation was done with the M06-2X//6-31G (d,p) density functional method in solvent conditions, using the polarizable continuum model (PCM), with a solvent dielectric constant of 40, intermediate between water and protein. The resulting energy difference between the flat and puckered ring configurations was just .4 kcal/mol. Based on the PDB survey and the low computed energy difference, we assigned a force constant of zero to the C6-C7-N8-C8A dihedral. The structure minimized with the force field [using the protX module of *Proteus* (Simonson, 2019)] then gave a planar geometry.

To test the DHF force field further, we performed MD simulations of DHF and the DHFR-DHF complex in solution. Simulation details and results are in Supplementary Material. When bound to the protein, DHF sampled both planar and slightly puckered geometries, with a mean C6-C7-N8-C8A dihedral angle of 11.1 ± 7.8° and a mean C7 distance from the ring plane of .2 ± .2 Å.

### 2.4 Ligand rotamers

#### 2.4.1 Ligand conformers

To identify favorable ligand conformations, we ran MD for DHF and TMP in explicit solvent, described by the TIP3P model (Jorgensen et al., 1983). Run lengths were 500 ns for DHF and 180 ns for TMP. The system temperature was controlled by Langevin dynamics at 300 K, with a friction coefficient of 1 ps^−1^. The pressure was kept constant at 1 atm using a Nose-Hoover Langevin piston (Feller et al., 1995), with a period of 50 fs. Electrostatic interactions were evaluated by the Particle Mesh Ewald method (Darden et al., 1993), using a cutoff distance of 12 Å. Van der Waals interactions were switched off at a cutoff distance of 12 Å. Simulations were done with the NAMD program (Phillips et al., 2005).

From the flexibility seen in the simulations, conformers were defined based on two soft, central dihedral angles and (for DHF) the Glu moiety, for which we used the 12 standard rotamers of the Tuffery library (Tuffery et al., 1991; Gaillard and Simonson, 2014). The soft dihedral angles are defined in Figure 1. Histograms from the MD simulations are shown in Supplementary Figure S2, with the selected conformers: 8 for DHF and 32 for TMP. Combining these conformations with those seen n the crystal and with the 12 Tuffery rotamers for the Glu moiety of DHF gave 97 rotamers for DHF and 33 for TMP.

#### 2.4.2 Docking the ligands within the DHFR active site

Each ligand conformer was then placed in the DHFR active site, where all side chains were mutated to alanine to maximize space for the ligand. The initial placement was done by fitting each conformer with respect to atoms of the native conformation close to the reaction site (atoms C6, N5, and C4A in DHF and atoms C4, C5, and C7 in TMP). Then, we performed 100 steps of minimization with harmonic restraints on the dihedrals that defined each conformer, using a force constant of 200 kcal/mol/rad^2^ and a tolerance range of ±5° around the initial angle. Calculations were done with the protX module of *Proteus* (Simonson, 2019). During minimization, the ligand and all atoms of DHFR residues within 5 Å of it were allowed to move, while the rest of the system was kept fixed. Solvent was described implicitly, with the GBLK model used below for the design stage. The conformers that, after minimization, did not have clashes with the protein were chosen to define the ligand rotamers (83 DHF and 33 TMP rotamers).

### 2.5 Protein structure

The protein was modeled in its apo and two holo states. The apo system consisted of *Escherichia coli* DHFR with the NADPH cofactor. In the two holo systems, either DHF or TMP was added to the apo state. We used the DHFR:NADP:DHF crystal [PDB code 1RX2 (Sawaya and Kraut, 1997)] for the DHF complex and the apo state. For the TMP complex, we used a crystal complex [PDB code 6XG5 (Manna et al., 2021)].

All DHFR histidines were set to be singly-protonated, except for H114 which was doubly-protonated, according to a neutron structure [PDB code 4PDJ (Wan et al., 2014)] determined at neutral pH. For DHF, we selected the tautomer with a protonated N3, two hydrogens on NA2, and a protonated N5, for a total charge of −1. For TMP, we selected the N1-protonated form, with a total charge of +1 (Figure 1). For NADPH, we selected the form with a fully-deprotonated terminal phosphate, with a total charge of −4.

### 2.6 MMGBLK energy function

Energy was computed using the MMGBLK energy model (Michael et al., 2017):

$$E=E_{\text{MM}}+E_{\text{GB}}+E_{\text{LK}}.$$ (4)

The MM term used the Amber ff99SB protein force field (Cornell et al., 1995) and ligand parameters derived here. For GB, we used the Native Environment Approximation (NEA), where the solvation radii of each residue were computed with the rest of the system in its native sequence and conformation (Mignon et al., 2020). The protein dielectric constant was 6.8, which is optimal with the LK model (Michael et al., 2017). The other LK parameters were reported earlier (Michael et al., 2017).

### 2.7 Unfolded state

The unfolded state energy *E* ^uf^ was estimated as a sum over residues and depends only on the amino acid composition of the sequence:

$$E^{\text{uf}}\left(S\right)=\underset{i\in S}{\sum }E^{\text{uf}}\left(t_{i}\right),$$ (5)

where the sum is over all positions of sequence *S* and *E* ^uf^(*t*_*i*_) is the unfolded energy of type *t* at position *i*, estimated using a tri-peptide model. Specifically, for each mutating position, chemical type and rotamer, we computed the interaction between the sidechain with itself and the adjacent backbone. Then for each chemical type, we collected the energy of the best rotamer at each position, and averaged over all positions to obtain *E* ^uf^(*t*_*i*_).

### 2.8 Choice of mutation space

The 20 DHFR residues closest to the DHF substrate were considered for redesign: 5I, 7A, 19A, 22W, 23N, 28L, 29A, 30W, 31F, 32K, 35T, 36L, 49S, 50I, 52R, 54L, 94I, 100Y, 113T, 153F. All residue types were allowed except Gly and Pro. Non-mutating residues between 5 and 10 Å from the binding site did not mutate but could change rotamers, chosen from the Tuffery library (Tuffery et al., 1991; Gaillard and Simonson, 2014), extended to include the orientations encountered in the PDB structure (native rotamers). Both ligands could adopt different rotamers (conformers and poses). The rest of the system, including the DHFR backbone, NADPH and all Gly and Pro residues, kept the positions they had in the experimental structure.

### 2.9 Calculation of the interaction energy matrix

For fast MC exploration, we precomputed the interactions between all pairs of residues, for all side chain types and rotamers, and stored them in an Interaction Energy Matrix (IEM), as detailed earlier (Gaillard and Simonson, 2014; Simonson, 2019).

### 2.10 MC protocol

The total bias potential *E* ^Bias^ at time *t* is given by:

$$E^{\text{Bias}}\left(s_{1}\left(t\right),s_{2}\left(t\right),\ldots ,s_{p}\left(t\right);t\right)=\underset{i}{\sum }E_{i}^{\text{Bias}}\left(s_{i}\left(t\right);t\right)+\underset{i<j}{\sum }E_{ij}^{\text{Bias}}\left(s_{i}\left(t\right),s_{j}\left(t\right);t\right),$$ (6)

where the first sum is over single positions, the second is over pairs, and *s*_*i*_(*t*) is the side chain type at position *i*. Single position and pair biases were updated at regular time intervals; at each update, the corresponding state of the system was penalized by adding a single-position $e_{i}^{B}\left(s_{i}\left(t\right);t\right)$ , or pair increments $e_{ij}^{B}\left(s_{i}\left(t\right),s_{j}\left(t\right);t\right)$ to the current bias potentials. The increments decreased exponentially over time (Villa et al., 2018):

$$e_{i}^{B}\left(s_{i}\left(t\right);t\right)=e_{0}\exp\left[-E_{i}^{\text{bias}}\left(s_{i}\left(t\right);t\right)/E_{0}\right],$$ (7)

$$e_{ij}^{B}\left(s_{i}\left(t\right),s_{j}\left(t\right);t\right)=e_{0}\exp\left[-E_{ij}^{\text{bias}}\left(s_{i}\left(t\right),s_{j}\left(t\right);t\right)/E_{0}\right],$$ (8)

where *e*_0_ and *E*_0_ are constants, *e*_0_ = .2 kcal/mol and *E*_0_ = 40 kcal/mol.

To optimize the bias, we did MC simulations with bias updates every 1000 MC steps. At first, the biases were optimized using only single-position terms. Two-position terms were added in cases where the single-position biases were not sufficient to flatten the landscape. In both adaptation and production MC simulations, we used a 10:1 ratio of conformation/sequence moves and included moves at two positions. Simulations were run at 300 K for 20 × 10^6^ steps in single-position designs and 10^8^ steps otherwise.

### 2.11 Amino acid classes for coupling analysis

To simplify the analysis of couplings between amino acid pairs, we grouped amino acid types into ten classes and computed couplings that were averaged over classes. The classes were: WF, Y, H, RK, ED, NQ, IVL, M, C, and AST.

## 3 Results

### 3.1 Mutating single positions

**FIGURE 2:**
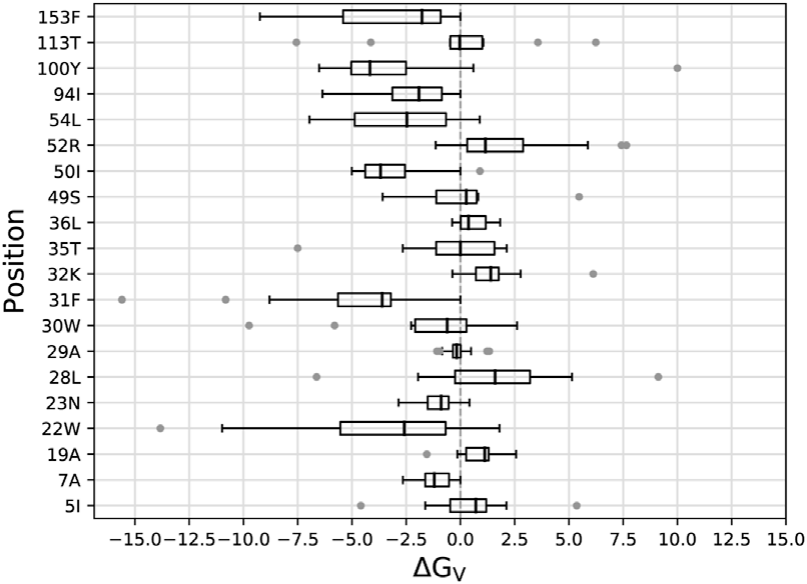
Box plots of vitalities (kcal/mol) from singleton redesign of 20 DHFR positions. Each box encloses half the data; the thick line is the median. Gray points are outliers more than 1.5 times the width of the second or third quartile. Whiskers are delimited, on each side, by the last point that is not an outlier.

We began by mutating, or “redesigning” the 20 residues closest to the DHF substrate, one residue at a time. For each position, we performed the bias adaptation stage, such that the sequence space was progressively flattened. Side chain types that could not fit sterically were manifested by bias values that increased to more than 20 kcal/mol, and were then excluded from the exploration space. Excluded types are shown in Table 1 for each ligand state: apo, DHF- or TMP-bound. After the flattening, we ran further, “production” MC, from which we obtained the biased populations and the stability, affinity and vitality changes (Eqs. 1, 3). Figure 2 shows the distribution of vitalities at each redesigned position. Ten positions had favorable vitality gains, of 1–4 kcal/mol on average. Vitality was mostly lost (more positive values) upon mutations at positions 19A, 32K, 36L, 52R, and 113T. A few very large vitality gains were due to loss of TMP binding through steric exclusion.

**TABLE 1:** Residue types excluded from the mutation space for singleton design.

Position	Apo	DHF	TMP
5I	RFW	RFWY	RFWY
7A	FWY	RHILKMFWY	RHILKMFWYV
19A	-	-	-
22W	-	-	-
23N	-	-	-
28L	-	-	-
29A	-	-	-
30W	-	-	-
31F	-	I	RIW
32K	-	-	-
35T	HFWY	RHKMFWY	RQHIKMFWY
36L	-	-	-
49S	RQIL	RQILKW	REHILKMFWYV
50I	W	FWY	FWY
52R	-	-	-
54L	-	W	W
94I	W	RFWY	RHLKFWY
100Y	RW	RW	IWV
113T	HFWY	RHKFWY	HWY
153F	-	-	W

Experimental resistance mutations from different studies are listed in Supplementary Table S2. Table 2 lists those that correspond to the active site positions redesigned here. Of seven such mutations, five had predicted vitality gains in the simulations. For the other two resistance mutations, the simulations predicted a loss of vitality. Either errors in the simulation model produced two false negatives, or the experimental resistance was not due to a vitality gain. Overall, it appears the simulations can help identify true positives, albeit not with 100% success.

**TABLE 2:** Known resistance mutations involving active site positions.

Mutation	Exp. vitality changes	Comp. vitality changes
L28R	+	−
W30R	+	+
F153S	+	+
W30C	+	+
W30Y	+	−
F153V	+	+
F153L	+	+

Symbols +, − denote changes of magnitude ≤1 kcal/mol.

**FIGURE 3:**
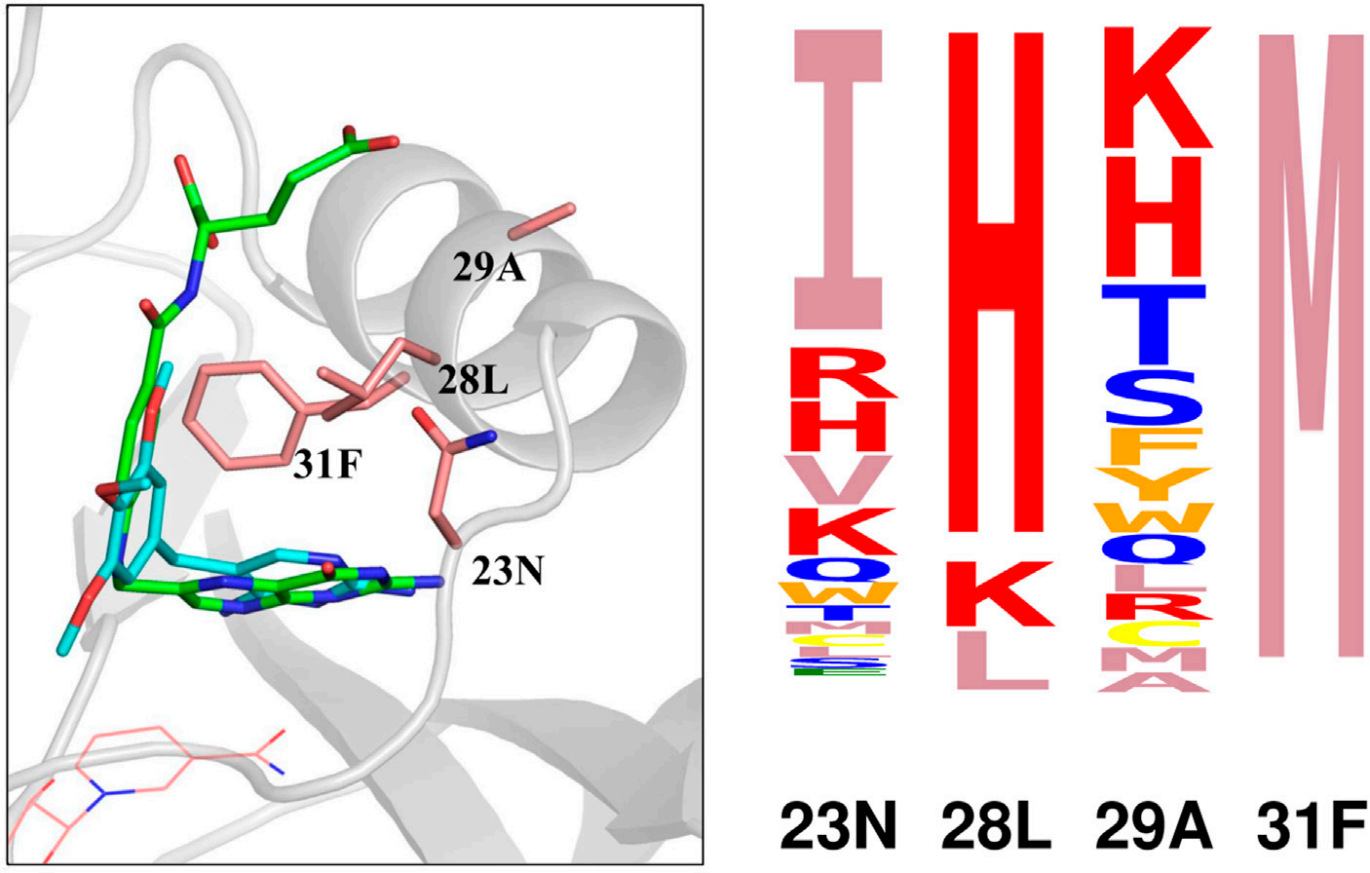
(Left) Closeup of four positions (pink sticks) in the DHFR complex with DHF (green sticks) and TMP (blue sticks). NADPH is shown as pink lines. (Right) Vitality logo from singleton redesign of the four positions.

To interpret the simulation results further, we focussed on four positions that form a cluster near the ligand site: 23N, 28L, 29A, and 31F. A structural view is in Figure 3. Detailed results are in Supplementary Table S3: stability, affinity and vitality values for each type at each position. The vitality profile for each position is represented by the logo in Figure 3. Based on vitality, position 23 prefers I, position 29 prefers K or H, position 28 prefers H, while position 31 overwhelmingly prefers M. At positions 23 and 29, there were modest vitality gains for several types, arising from small gains in DHF binding, by .4–.6 kcal/mol at most, associated with somewhat larger losses of TMP binding, by .7–1.7 kcal/mol. At positions 28 and 31, a few large side chain types produced much larger losses of TMP binding, by 4–8 kcal/mol. There were also two very large losses of TMP binding, for F31M and F31K, and one very large loss of DHF binding, for L28I. We hypothesized that the largest losses of TMP binding were largely due to the rigid backbone, discrete rotamer approximations made in the simulations. This was confirmed by MMGBLK binding calculations (Michael et al., 2017), detailed in Supplementary Table S4.

### 3.2 Mutating pairs of positions

We now turn to the mutation, or redesign of pairs of residues in the active site. During each MC simulation, two positions could mutate simultaneously, giving each more flexibility to change its type. We considered 51 pairs, formed by the 20 positions above, such that the distance between the 2 *C*_*β*_ atoms was below 10 Å. From the 51 redesigns, 2657 sequences were predicted to cause a vitality gain. 28 pairs, listed in Table 3, gave at least one hit with a vitality gain, while 16 produced gains over 3 kcal/mol.

**TABLE 3:** Free energies (kcal/mol) from the top hits at the top 28 pairs.

Pair	Nat	Seq	Δ*G*_Vit_	$\Delta G_{\text{Bind}}^{\mathrm{DHF}}$	$\Delta G_{\text{Bind}}^{\mathrm{TMP}}$	$\Delta G_{\text{Fold}}^{\mathrm{Apo}}$
94 100	IY	MN	−11.8	1.4	13.2	−2.2
23 28	NL	IF	−9.4	1.4	10.8	0.7
7 153	AF	AI	−9.3	−0.8	8.5	−1.6
50 94	II	MC	−7.8	2.4	10.2	−0.1
52 54	RL	CM	−7.7	0.6	8.3	1.1
32 54	KL	RM	−7.1	0.1	7.2	1.1
36 54	LL	IM	−6.7	0.0	6.7	0.4
35 54	TL	TM	−6.6	0.1	6.7	1.6
49 50	SI	CM	−6.4	1.7	8.1	−0.1
5 100	IY	VD	−6.4	−1.5	4.9	0.6
50 52	IR	MC	−6.1	2.4	8.5	−1.0
29 31	AF	KL	−4.7	0.4	5.1	1.0
31 32	FK	QR	−4.2	−3.1	1.2	1.6
31 113	FT	FV	−4.1	0.2	4.3	2.0
31 36	FL	QI	−4.0	−3.3	0.7	1.0
7 31	AF	AC	−3.7	−4.1	−0.5	1.3
28 32	LK	HR	−1.7	1.1	2.9	1.3
29 32	AK	HR	−1.5	−0.5	1.0	1.1
19 49	AS	AC	−1.4	−0.1	1.3	0.3
7 28	AL	AH	−1.3	0.9	2.2	1.8
28 113	LT	HT	−1.2	1.0	2.2	1.8
28 29	LA	HA	−1.2	1.0	2.2	1.8
5 35	IT	VT	−0.6	−0.1	0.5	1.3
5 113	IT	VT	−0.6	−0.1	0.5	1.3
5 7	IA	VA	−0.5	0.0	0.5	1.3
32 36	KL	RK	−0.5	−0.2	0.3	0.8
35 36	TL	TK	−0.4	−0.3	0.1	1.2
32 35	KT	RT	−0.4	0.1	0.5	−0.4

**FIGURE 4:**
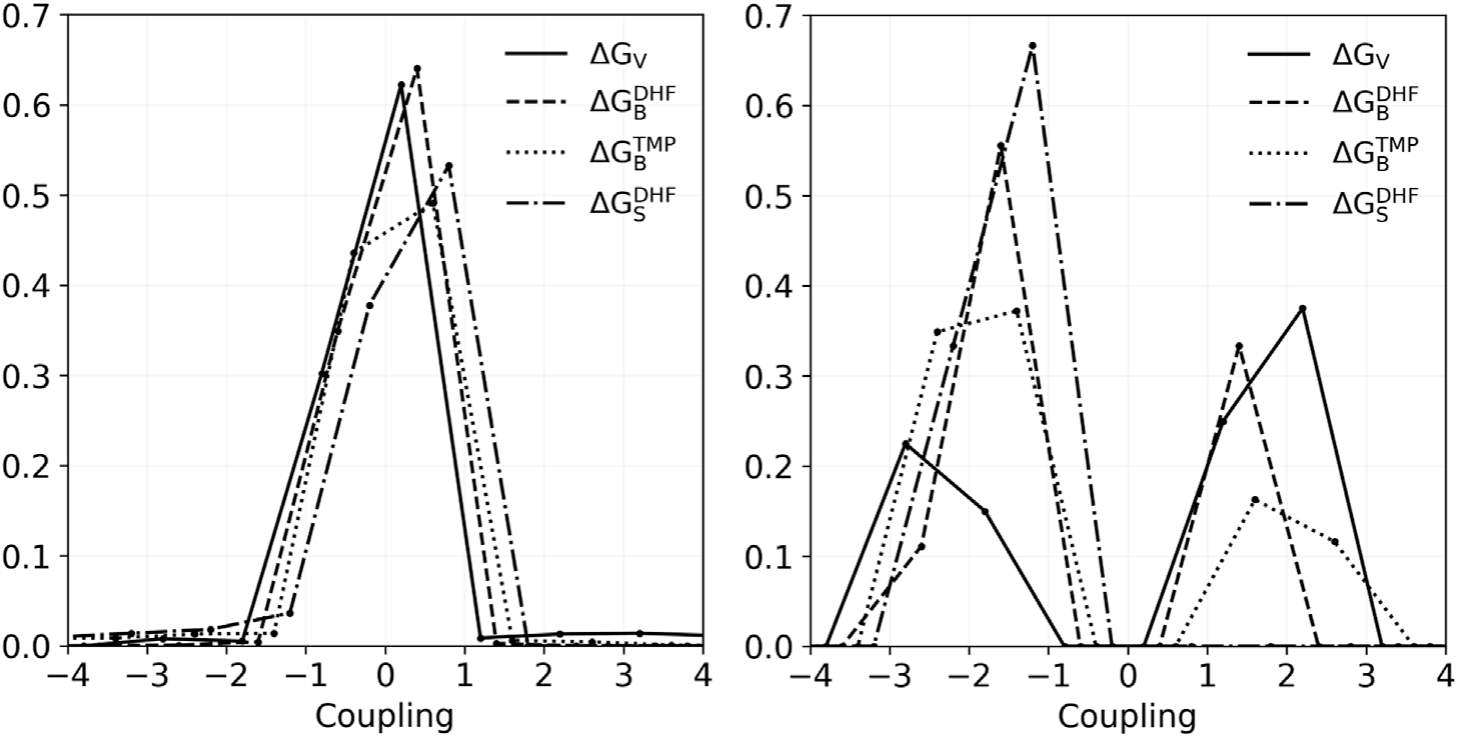
Histograms of coupling values for vitality, DHF binding, TMP binding, and stability of the 1149 class combinations that occurred in the redesign of 51 pairs. Left: All couplings. Right: couplings in the range 1–3 kcal/mol.

Also of interest are the couplings between pairs of positions *I*, *J*, defined as

$$C_{IJ}\left(t,t^{\prime }\right)=\Delta G_{IJ}\left(t,t^{\prime }\right)-\Delta G_{I}\left(t\right)-\Delta G_{J}\left(t^{\prime }\right),$$ (9)

where *t*, *t*′ are the side chain types, the first free energy was from the pair redesign, and the others from the two singleton redesigns. They can be the folding free energies, binding free energies, or a DHF/TMP binding free energy difference, in other words a vitality. To simplify the analysis, we grouped amino acid types into 10 classes, and averaged couplings within classes (see Methods). Figure 4 depicts histograms of couplings for all 51 pairs and the four properties: folding, DHF binding, TMP binding, and vitality. In all, there were 1149 pairs of types and couplings for each property. Most values were less than 1 kcal/mol: 99.0% for DHF binding, 92.7% for TMP binding and 92.4% for vitality. The largest negative coupling overall was −9.1 kcal/mol, for the TMP binding of IVL-IVL classes of the pair 30–153. The largest positive coupling was 10.6 kcal/mol, for the vitality of the same pair and classes. If we exclude those extreme cases, the other couplings were less than 4 kcal/mol in absolute magnitude.

As above, we considered in more detail the results for positions 23, 28, 29, and 31. Four pair redesigns were done for these positions. Supplementary Table S5 lists the top 15 vitality variants for these four pairs, along with the DHF and TMP binding free energies and the apo-protein folding free energy. The only couplings greater than .5 kcal/mol (in absolute magnitude) involved the pair 28–31, and variants with F at position 28. We saw above that this residue type led to an exaggerated steric repulsion with TMP in the singleton design. Here, by mutating position 31 simultaneously, the steric repulsion was alleviated somewhat. Other than these cases, the pair designs recapitulated the singleton designs, described above, and simply added together the contributions of each residue in the pair (up to a very small coupling correction). Disregarding the variants with L28F, vitality gains were in the range 2–5 kcal/mol for the pairs 23–28, 28–31, and 29–31. The 28–29 pair was less effective, with vitality gains of 1.2 kcal/mol at most.

### 3.3 Mutating the quartet 23–28–29–31

We chose the positions 23–28–29–31 to illustrate the mutation of four positions at once, or quartet redesign. These positions are close together in the active site (Figure 3) and might be expected to have significant couplings. There are several ways to define couping within a quartet, even for a single physical quantity such as vitality. The simplest considers the quartet as a group of two pairs and compares the quartet result to the sum of two pair results. Here, since 28–31 have the strongest coupling, we considered the two pairs 28–31 and 23–29. We denote the coupling by *C*_*QP*_ (P for pair).

**FIGURE 5:**
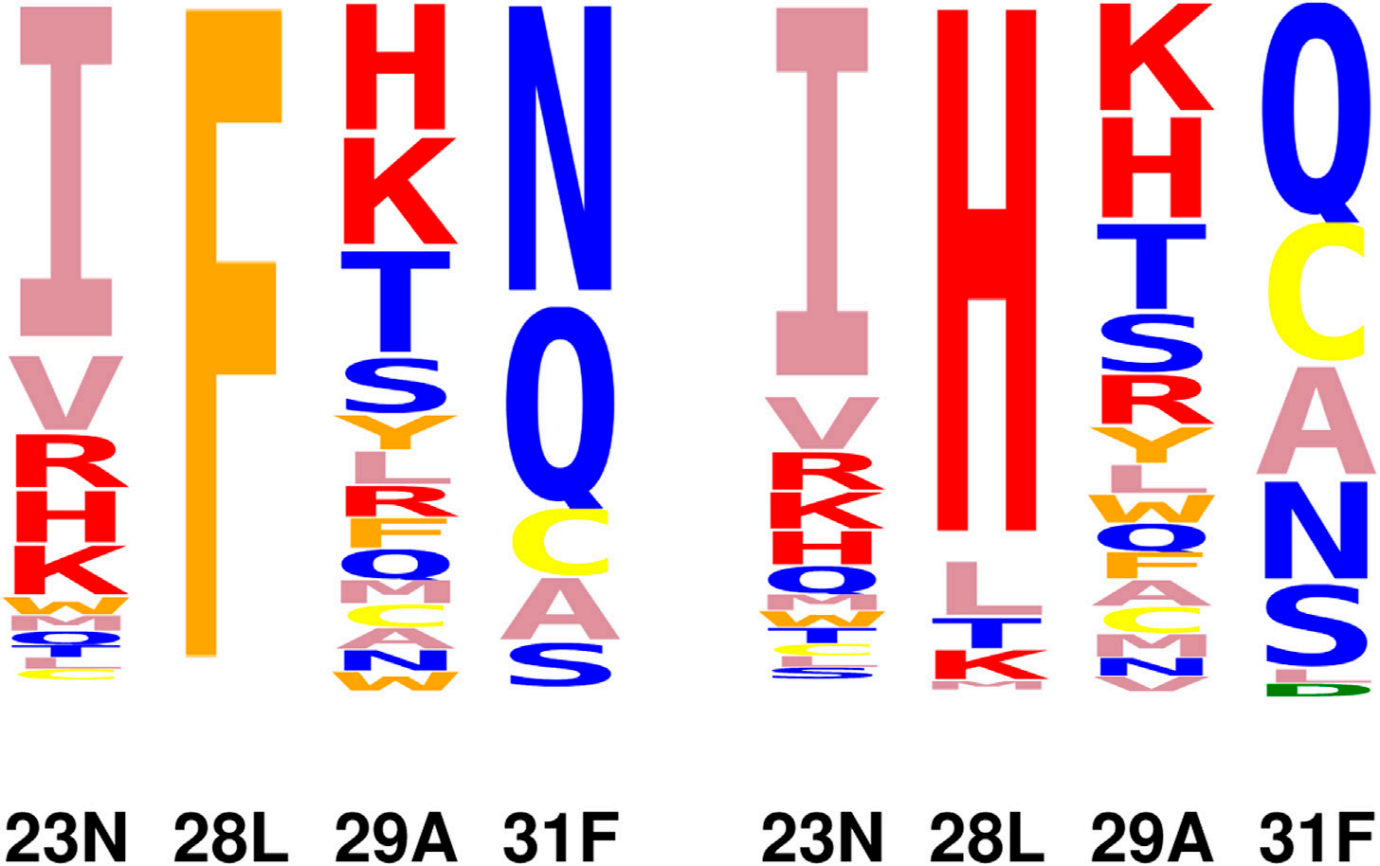
Vitality logos from quartet redesign. Right: variants with F28 excluded.

From the redesign of the quartet, 20064 variants were predicted to have improved vitality, compared to the native. We noted above that variants with Y or W at position 28 or E, M, Y, H, K at position 31 displayed an exaggerated loss of TMP binding because of the rigid backbone, discrete rotamer approximations. Excluding these variants, there were 17369 variants with a predicted vitality gain. Sequence logos with and without F at position 28 are shown in Figure 5. Vitality, affinity, folding free energies, and couplings are given in Table 4 for the top 10 variants (ranked by vitality). Fairly large couplings are seen for variants with H at position 28, which reflect a decrease in steric exclusion of TMP when position 28 is mutated in combination with its closest neighbors. Aside from these cases, the quartet redesign mostly recapitulates the pair and singleton redesigns above. The favorable vitality effects seen at positions 23, 28, and 31 are roughly additive.

**TABLE 4:** Top variants from quartet design of positions 23–28–29–31.

Seq	Δ*G*_Vit_	$\Delta G_{\text{Bind}}^{\text{DHF}}$	$\Delta G_{\text{Bind}}^{\text{TMP}}$	$\Delta G_{\text{Fold}}^{\text{apo}}$
IHKQ	−9.4 (−.2)	−2.5 (.4)	6.9 (.6)	3.0 (−.7)
IHSQ	−9.2 (−.5)	−2.1 (.3)	7.1 (.8)	3.3 (−.6)
IHTQ	−9.0 (−.1)	−2.0 (.3)	7.0 (.4)	2.9 (−.4)
IHRC	−9.0 (−1.0)	−3.8 (.0)	5.2 (1.1)	2.7 (−.4)
IHRQ	−8.9 (−.7)	−2.9 (.0)	6.0 (.6)	4.0 (.1)
IHHQ	−8.8 (.2)	−1.6 (1.4)	7.2 (1.1)	3.8 (−.6)
IHHC	−8.8 (−.1)	−3.7 (.2)	5.1 (.2)	3.8 (.1)
IHAQ	−8.8 (−.7)	−1.9 (.6)	6.9 (1.3)	2.0 (−1.0)
IHKS	−8.7 (−.2)	−4.0 (.4)	4.7 (.6)	3.8 (−.2)
IHHA	−8.6 (−.1)	−4.3 (.1)	4.3 (.2)	3.5 (−.1)

Top vitalities, with DHF, and TMP, binding free energies and apo-protein folding free energies (kcal/mol). Couplings in parentheses. Variants with WYF, at position 28 omitted.

## 4 Concluding discussion

Experimental methods to identify resistance mutations are mostly low- or medium-throughput (Jackel et al., 2008; Thompson et al., 2020), and do not usually reveal the underlying resistance mechanism. Predicting them with simulations is another goal, and one route was proposed here. We focussed on the situation where an antibiotic inhibits an enzyme, and resistance arises from changes in the inhibitor and substrate binding. These changes were captured by the enzyme “vitality”. Resistance mutations should also maintain transition state binding, and indeed, the original definition of vitality was based on the relative binding of the inhibitor and the transition state (Gulnik et al., 1995; Ishikita and Warshel, 2008; Singh et al., 2012; Jindal et al., 2017). However, substrate binding is much simpler to model. It does not involve a determination of the transition state, which might require a quantum mechanical study of the entire reaction pathway. We can use substrate binding as a proxy for activity, if we are willing to speculate that many mutations that maintain ground state binding will also maintain transition state binding.

We borrowed methodology from CPD. In particular, adaptive landscape flattening (Villa et al., 2018) allows one to score hundreds of thousands of sequences according to substrate and inhibitor binding, and thus vitality. We used an established CPD model, with a molecular mechanics energy, a continuum solvent, a fixed protein backbone and a discrete rotamer library (Mignon et al., 2020). It gave good accuracy recently for several ligands binding to several dozen variants of the methionyl-tRNA synthetase enzyme (Opuu et al., 2020). However, the method could also be used with other models or energy functions, such as knowledge-based functions.

The DHFR simulation model included new DHF and TMP force field parameters in several tautomers and protonation states, and a rotamer library for each ligand, which are all available and of general interest. We then considered mutations at 20 positions in the DHFR active site, which span a vast mutation space of over 10^26^ possible variants. We did not attempt to mutate 20 positions at once, since adaptive landscape flattening is effective for at most four to five positions mutating together. Also, known DHFR resistance mutations involve only one or a few positions at a time (Supplementary Table S2). We adopted a stepwise, hierarchical approach, where small groups of positions were studied first. Two small groups can then be combined to form a larger group. If couplings between the two subgroups are small, the mutation space of the large group can be accurately represented by combining mutations of the subgroups. Thus, we showed that mutations of the quartet 23–28–29–31 were accurately described by combining mutations of the 23–29 and 28–31 pairs. From 20 active site positions, one can form 4845 quartets, which encompass over one billion possible sequences. Once the pairs have been redesigned, most of these quartets are well-described by combining underlying pairs. Thus, our method can sample the full space of quartet sequences, if one is willing to accept errors for a tiny percentage of quartets that are not well-approximated by pairs. The total computational time to explore this space is a few days on a single desktop computer. The hierarchical sampling approach could be of general interest for CPD.

The small, predicted, direct pair couplings suggest that higher order couplings are even smaller, and are well-approximated by combinations of pairwise couplings. Indirect couplings can of course exist, for example when two distant positions are both involved in the binding of a large substrate. Nevertheless, the small direct couplings suggest that over the course of evolution, resistance mutations at multiple positions can often occur sequentially, and do not need to appear simultaneusly.

There are seven known resistance mutations involving active site positions. Five were recovered here, with large predicted vitality gains. Another, L28R, was not highly ranked (Table 2), but the homologous mutant L28H was among the top predictions (Supplementary Table S3). Out of the 20 positions redesigned, half gave mean vitality gains, in the range 1–4 kcal/mol. Analyzing a small cluster of illustrative positions, we observed many vitality gains produced by TMP binding losses, often due to steric exclusion of the ligand. DHF binding gains were less common and smaller, around .5–1 kcal/mol in favorable cases, relative to the wildtype binding. As expected, the wildtype sequence is well-optimized for substrate binding, and there are not many variants that do better.

Combining pair hits from Table 3, we obtain quartets with very large vitality gains. Thus, there is a large reservoir of mutations that can be tapped to increase vitality. Notice, however, that because wildtype TMP binding (nanomolar) is much stronger than DHF binding (micromolar), and TMP concentrations *in vivo* (mM) are much larger than DHF concentrations (*μ*M), large vitality gains (around six log units, or 8 kcal/mol) are needed to reverse the binding preference and fully reestablish enzyme function. Notice also that many of the vitality gains are due to TMP exclusion. However, once TMP binding has been reduced to a level that is below DHF binding, further losses may not impact the actual bacterial fitness. Thus, vitality gains much greater than six log units are probably not useful in practice.

In conclusion, we have proposed a new, computational method to predict a simple class of resistance mutations. For DHFR, we have recovered most of the known active site resistance mutants and predicted others. We used a powerful adaptive landscape flattening and a hierarchical sampling of positions in the active site to overcome the combinatorial complexity of the problem. The method can be extended in several ways; for example, transition state binding could be considered instead of substrate binding. While more experimental validation is needed, we expect the method can already make predictions and help guide experimental exploration of enzyme fitness and resistance.

## Data Availability

The original contributions presented in the study are included in the article/Supplementary Material, further inquiries can be directed to the corresponding author.
