# Supplementary material for: Computational protein design repurposed to explore enzyme vitality and help predict antibiotic resistance
Source: Front Mol Biosci. 2023 Jan 9;9:905588. doi: 10.3389/fmolb.2022.905588 (PMC9868620; doi:10.3389/fmolb.2022.905588)
Supplement: Supplementary file 1 [file DataSheet1.PDF]

Supplementary Material:
Computational protein design repurposed to explore enzyme vitality and help predict antibiotic resistance

Eleni Michael, Rémy Saint-Jalme, David Mignon and Thomas Simonson

We begin by detailing methods used for the molecular dynamics (MD) simulations of DHFR. We then describe the methods used to survey PDB structures, in order to evaluate the interactions of the NADPH terminal phosphate and the geometry of the DHF pterin ring. We then report supplementary results. We first report interactions of the NADPH terminal phosphate seen in the PDB and MD simulations (Table SM1). Next, we report the geometry of the DHF pterin ring from the PDB and MD (Fig. SM1). These results help determine the preferred NADPH protonation state and the DHF force field parameters. We also report DHF conformers seen in MD simulations (Fig. SM2). We then recapitulate *E. coli* DHFR resistance mutations from the literature (Table SM2). Table SM3 gives details from the redesign of four selected positions in the active site: 23N, 28L, 29A, 31F. DHF and TMP binding free energy changes are reported along with vitality and folding free energy changes. Table SM4 gives the results of MMGBLK calculations for TMP binding to selected mutants, confirming that some of the large values in the main text are artefacts of the fixed backbone approximation. Finally, Table SM5 reports additional details from the redesign of selected pairs in the active site: 23–28, 28–29, 28–31, and 29–31. These are the pairs that form the redesigned quartet 23-28-29-31 (see main text). Force field parameters for DHF and TMP are given in separate files: DHF.data and TMP.data.

1 Methods

1.1 MD simulations of DHFR

The NADPH terminal phosphate can be either singly-protonated (-1 charge) or fully-deprotonated (-2 charge). We did MD simulations of the DHFR-DHF-NADPH complex with NADPH in either of these protonation states. System preparation for MD was done with the protX module of Proteus [1]. Initial coordinates were taken from the X-ray structure with PDB code 1RX2 [2]. Hydrogen atoms were positioned using the hbuild facility and subjected to 40 steps of conjugate gradient minimization, with everything else

frozen. The complex was then positioned in an equilibrated, cubic water box. Explicit water was described by the TIP3P model [3]. Protein was described by the Amber ff14SB force field. NADPH parameters developed by Ulf Ryde [4, 5] for both protonation states were formatted for protX and NAMD. Minimization, equilibration and production phases were conducted with the NAMD program [6]. The solvated system was initially minimized through 50000 steps of the conjugate gradient algorithm, using harmonic restraints for heavy atoms, with force constants related to the B-factor in the X-ray structure. The system was then equilibrated by a 1 ns long simulation with a 2 fs time-step in the NVT ensemble at 300 K, with the same harmonic restraints. Production simulations were performed without restraints, in the NPT ensemble, for a total duration of 300 ns. The system temperature was controlled by Langevin dynamics at 300 K, with a friction coefficient of 1 ps^{-1}. The pressure was kept constant at 1 atm using a Nose-Hoover Langevin piston [7, 8], with a period of 50 fs. Electrostatic interactions were evaluated by the Particle Mesh Ewald method [9], using a real space cutoff of 12 Å. Van der Waals interactions were switched off at a cutoff of 12 Å.

1.2 Survey of PDB structures

To obtain statistics on the PDB environment of the NADPH terminal phosphate, 61 experimental structures of DHFR in complex with NADPH were collected, using the advanced PDB search tool [10], searching for DHFR from *E. coli* in complex with $NADP^+$ or NADPH. The query was: "Uniprot molecule name = 'Dihydrofolate reductase', Chemical component with chemical ID = 'NAP' ($NADP^+$) or 'NDP' (NADPH)." The experimental structures were then used to count the number of terminal phosphate oxygen atoms engaged in hydrogen bonds with DHFR residues. To obtain statistics on the geometry of the pterin ring moiety of DHF, 30 experimental *E. coli* DHFR structures were collected from the PDB, containing as a ligand either DHF or folate (FOL).

2 Results

2.1 NADPH phosphate interactions in PDB and simulations

The NADPH terminal phosphate can be either singly-protonated (-1 charge) or fully-deprotonated (-2 charge). To determine its preferred state in DHFR, we analyzed its hydrogen bond interactions over the course of MD simulations with either protonation

state, as well as those found in PDB structures of DHFR. Results are in Table SM1. From the PDB structures, we deduced the number of phosphate oxygens forming hydrogen bonds with the four nearby DHFR residues: R44, S63, S64 and Q65. We also report the corresponding interatomic distances in the 61 experimental structures. In most PDB structures, two phosphate oxygens engage in hydrogen bonds with R44, whereas one engages with S63 and S64. In just 11 structures, the phosphate oxygens are within hydrogen bonding distance of Q65, with one oxygen usually involved.

MD simulations of the complexes were done with NADPH in either protonation state. The frequency of hydrogen bond formation is reported in Table SM1 from each simulation. With both protonation states, R44 always forms a salt bridge with the phosphate, with very similar statistics. However, only with the -the 2 phosphate charge does the simulation maintain hydrogen bonds to S64 (at least one 100% of the time) and to S63 (76% of the time). The S63 interaction is not quite as prevalent as in the PDB (59 out of 61 structures). Nevertheless, agreement with the experimental structures is distinctly better when the system is simulated with a fully-deprotonated NADPH terminal phosphate. This is fully consistent with the picture seen in the neutron structure (see main text) [11].

Table SM1: Hydrogen bond statistics for the NADPH phosphate from the PDB and MD

	MD simulations Protonation state[a]		PDB structures Oxygen no.[b]				
Residue	-2	-1	0	1	2	3	Distance[c]
R44 (N*)	308	306	6	3	52	0	2.8 (0.4)
S63 (OG)	76	10	2	58	1	0	2.6 (0.1)
S64 (OG)	151	71	0	53	8	0	2.7 (0.1)
Q65 (NE2)	7	2	50	8	3	0	5.2 (1.1)
Q65 (OE1)	0	0	53	8	0	0	5.0 (0.8)

Hydrogen bond distance cutoff between heavy atoms is 3.5 Å.
[a]Hydrogen-bond frequency (%) between any phosphate oxygen and DHFR residue atoms, over the 300 ns MD simulations. Frequency is summed over the four phosphate oxygens and over the nitrogens in the case of R44.
[b]Number of experimental structures out of 61, in which 0, 1, 2 or 3 oxygens of the phosphate are within h-bond distance of the DHFR residue atoms.
[c]Average distance (standard deviation in parentheses) between the closest phosphate oxygen and the DHFR residue atoms in the 61 experimental structures.

2.2 DHF pterin ring pucker in PDB and MD simulations

Force field parameters for the DHF pterin ring pucker were obtained from MD simulations of the DHFR complex with DHF and from 30 PDB structures. Puckering was measured by the C6-C7-N8-C8A dihedral angle and the C7 distance from the ring plane. Figure SM1 shows the histograms obtained. In the experimental, PDB distribution, the density is concentrated around zero with small deviations, for both the dihedral angle and the C7 distance, indicating that the ring is mostly flat. The mean dihedral angle was 1.3±1.0°and the average C7 distance from ring plane was 0.04±0.03 Å. In the MD simulations, the distributions are broader, and encompass the experimental ones. The mean C6-C7-N8-C8A dihedral angle was 11.1±7.8°, and the mean C7 distance to the ring plane was 0.2±0.2 Å. Thus, in the simulations, the ring visits slightly puckered conformations, but for the most part, it is almost flat, similar to the experimental structures. This led us to retain the initial force field parameters for the ring puckering dihedral (zero force constant). We also computed the rms deviation of the DHFR backbone atoms, NADPH and DHF heavy atoms (a total of 710 atoms) with respect to the X-ray structure during the MD simulation. The average rms deviation was 1.2±0.2 Å. The low value indicates a good performance of the force field parameters.

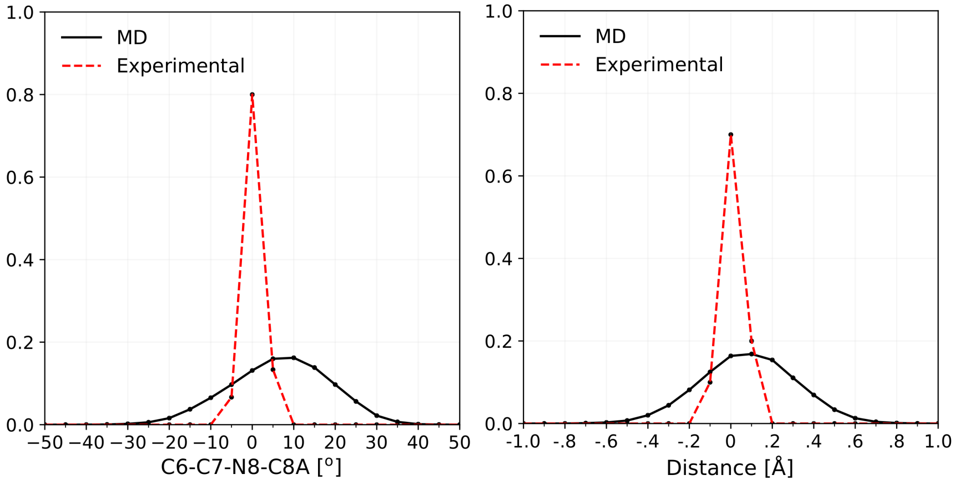

Figure SM1: Histograms of the C6-C7-N8-C8A dihedral angle (left) and the C7 distance from pterin ring plane (right) from 300 ns of MD (black solid line) or from 30 experimental structures (red dashed line).

2.3 DHF conformations from MD simulations

To identify favorable ligand conformations, we ran MD for DHF and TMP in explicit solvent. Run lengths were 500 ns for DHF and 180 ns for TMP. From the flexibility seen in the simulations, conformers were defined based on two soft, central dihedral angles, defined in the main text, Fig. 1. Fig. SM2 shows histograms from the MD simulations.

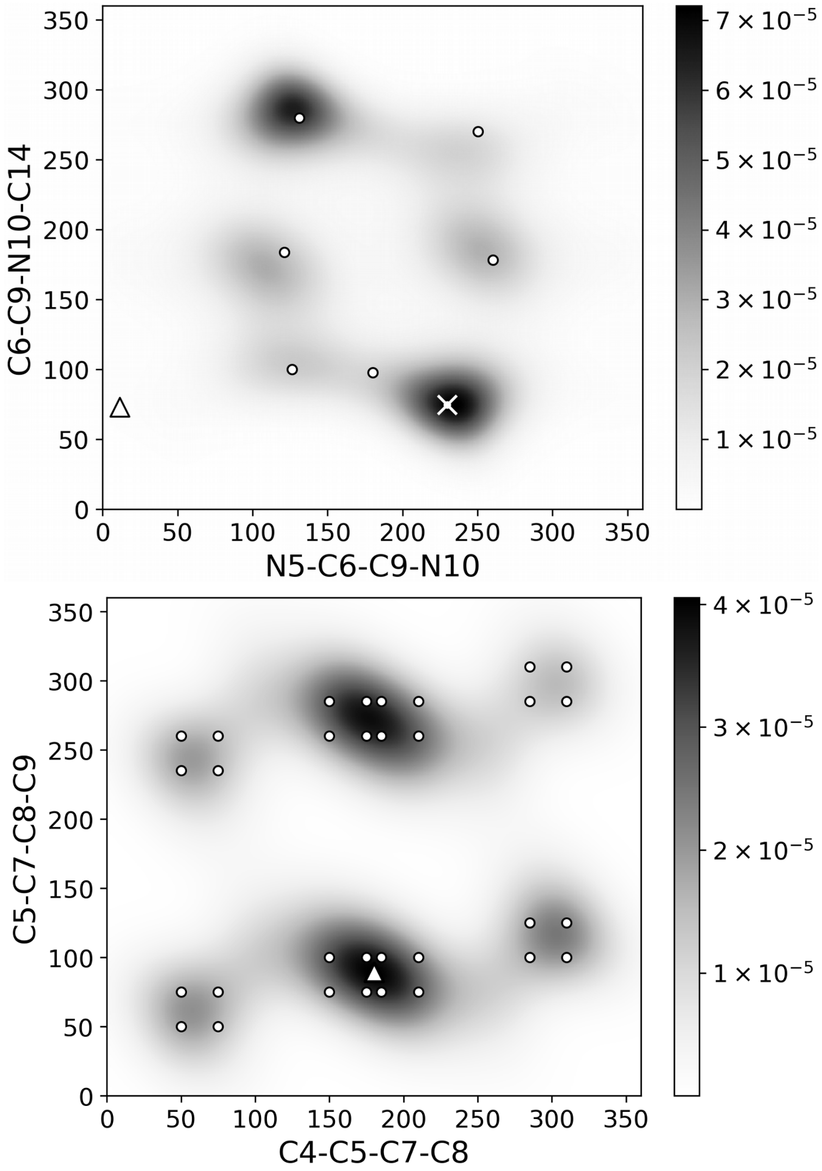

Figure SM2: Histograms of dihedral angles visited during the MD simulations of DHF (above) and TMP (below). Conformers selected for docking and rotamer construction are marked by circles or crosses; crosses indicate those rejected after docking. A triangle marks the native, crystal conformation from the DHFR complex.

2.4 Experimental resistance mutations for *E. coli* DHFR

Table SM2: *E. Coli* DHFR mutants that lead to TMP resistance

Mut.	K_M (μM)	k_{cat} (s^{-1})	K_I(nM)	Ref.
WT	0.806 ± 0.077	26.39 ± 0.94	9.1 ± 0.3	[14]
V10A	0.494 ± 0.021	368.81 ± 89.73	332.8 ± 81.0	
M20V	1.180 ± 0.077	65.04 ± 1.04	1096.7 ± 17.6	
A26T	0.327 ± 0.036	120.23 ± 8.08	166.2 ± 11.2	
W30R	0.272 ± 0.120	264.10 ± 0.31	132.5 ± 0.2	
H45R	0.501 ± 0.054	150.39 ± 3.37	618.4 ± 13.8	
I94L	2.060 ± 0.707	438.60 ± 14.38	160.9 ± 5.3	
WT	2.86 ± 0.24	5.36 ± 0.60	4.59 ± 0.76	[15, 16]
I5F	7.68 ± 1.04	3.78 ± 0.54	11.90 ± 2.70	
M20I	3.57 ± 0.19	7.69 ± 1.21	2.90 ± 0.45	
P21L	4.08 ± 0.49	3.19 ± 0.56	10.34 ± 0.43	
A26T	7.65 ± 1.10	3.70 ± 0.30	10.09 ± 1.94	
D27E	56.40 ± 5.25	14.31 ± 1.49	20.21 ± 3.11	
L28R	0.95 ± 0.09	1.13 ± 0.05	61.94 ± 4.73	
W30G	9.49 ± 2.12	8.18 ± 0.70	10.20 ± 1.22	
W30R	4.97 ± 0.30	8.62 ± 1.05	10.53 ± 2.42	
I94L	14.87 ± 1.44	7.71 ± 1.45	19.15 ± 2.99	
R98P	34.93 ± 4.65	2.82 ± 0.41	137.44 ± 14.57	
F153S	11.32 ± 3.29	5.62 ± 1.22	17.00 ± 2.28	
WT	0.81 ± 0.31	15.3 ± 0.2	0.165 ± 0.057	[17]
P21L	2.39 ± 0.26	24.2 ± 0.4	5.14 ± 0.72	
W30R	2.1 ± 1.2	1.36 ± 0.04	9.7 ± 5.4	
WT	1.49 ±0.14	8.16 ± 3.27	2.39 ± 1.06	[18]
L28R	0.62 ± 0.01	1.30 ± 0.01	24.63 ± 1.34	
P21Q, A26V, A26S, W30C, W30Y, F153V, F153L	resistance-conferring mutations observed in morbidostat experiments			[15, 16, 19]
P21L, W30R	were found in clinical isolates			[19–21]
P21L, A26T, W30R, I94L	were reported in laboratory selection			[14, 19]
L28R, W30C	appeared in selection experiments on agar plates			[19]

2.5 Singleton redesign of positions 23, 28, 29, 31

Table SM3: Free energies and vitalities (kcal/mol) from singleton design of four positions

Seq	ΔG_{Vit}	$\Delta G^{DHF}_{\text{Bind}}$	$\Delta G^{TMP}_{\text{Bind}}$	$\Delta G^{Apo}_{\text{Fold}}$	Seq	ΔG_{Vit}	$\Delta G^{DHF}_{\text{Bind}}$	$\Delta G^{TMP}_{\text{Bind}}$	$\Delta G^{Apo}_{\text{Fold}}$
		23N					28L		
I	-2.9	-0.1	2.8	-1.0	F	-6.6	1.6	8.2	1.7
R	-1.8	-0.5	1.3	-1.2	Y	-2.0	3.5	5.4	1.3
V	-1.8	-0.1	1.7	-0.9	W	-1.4	3.2	4.6	1.6
K	-1.8	-0.6	1.2	-0.9	H	-1.3	1.0	2.2	1.8
H	-1.7	-0.5	1.2	-1.4	K	-0.1	0.6	0.7	1.3
Q	-1.3	0.0	1.3	-0.2	L	0.0	0.0	0.0	0.0
W	-1.3	0.1	1.3	-0.4	T	0.2	1.1	0.9	0.8
T	-1.1	-0.2	0.9	0.0	M	1.3	1.2	-0.2	0.4
M	-1.0	-0.1	0.9	-0.5	R	1.6	1.7	0.1	0.3
C	-0.9	-0.1	0.8	0.0	S	1.9	1.6	-0.3	1.9
L	-0.9	-0.1	0.8	-1.0	C	2.0	1.4	-0.6	1.7
S	-0.8	-0.1	0.7	0.3	A	2.4	1.6	-0.7	1.1
E	-0.6	0.6	1.2	0.8	N	2.6	2.3	-0.3	2.0
Y	-0.5	0.0	0.5	-0.3	Q	3.8	3.6	-0.2	1.4
A	-0.5	-0.1	0.3	0.2	D	4.5	5.3	0.8	3.7
F	-0.1	0.0	0.1	0.1	V	4.7	4.2	-0.5	0.9
N	0.0	0.0	0.0	0.0	E	5.1	4.4	-0.8	2.4
D	0.4	0.6	0.1	2.4	I	9.1	9.4	0.2	0.4
		29A					31F		
K	-1.1	-0.4	0.7	0.7	M	-15.6	2.3	17.9	-0.4
H	-1.0	-0.5	0.5	1.5	K	-10.8	6.2	17.1	0.3
T	-0.8	0.1	1.0	0.3	E	-8.8	-1.6	7.3	3.7
S	-0.6	0.1	0.7	0.9	Y	-6.1	-1.9	4.2	4.8
F	-0.4	0.2	0.6	1.1	H	-5.5	0.2	5.7	0.6
Y	-0.3	0.1	0.5	1.1	Q	-4.0	-3.3	0.7	2.2
W	-0.2	0.2	0.4	1.2	C	-3.7	-4.3	-0.6	1.4
Q	-0.2	0.1	0.3	1.2	L	-3.6	0.8	4.4	0.3
L	-0.2	0.1	0.3	0.5	N	-3.5	-3.1	0.4	1.9
R	-0.2	-0.5	-0.3	0.9	A	-3.4	-4.7	-1.3	1.3
C	-0.1	0.1	0.2	0.9	S	-3.3	-4.9	-1.5	2.4
M	-0.1	0.1	0.2	0.7	D	-2.9	-3.1	-0.2	4.3
A	0.0	0.0	0.0	0.0	T	-1.3	-2.0	-0.7	1.0
N	0.0	0.2	0.1	1.3	V	-0.6	4.5	5.2	1.0
V	0.1	0.0	-0.1	2.2	F	0.0	0.0	0.0	0.0
I	0.5	0.1	-0.4	3.0					
E	1.2	1.0	-0.2	2.1		WRI		unsampled	
D	1.3	0.9	-0.5	3.0					

2.6 MMGBLK analysis of TMP binding

We performed MMGBLK binding calculations [22] for TMP binding to selected DHFR variants. The energy of the system was computed for the bound and unbound structures to obtain an affinity estimate. The unbound structure was obtained by simply separating the protein and ligand, without altering their conformations ("single structure" method). The calculations were done either with a very short energy minimization of the complex, or a longer minimization one. The short minimizations lasted 15 steps, as in the CPD IEM calculation. The longer minimizations lasted 100 steps, with both side chain and backbone flexibility. Results are in Table SM4. They confirm that, especially for position 31, the largest losses of TMP binding arise from steric conflicts that are eliminated when one relaxes the rigid backbone and discrete rotamer approximations. Nevertheless, with the MMGBLK binding calculations, the mutations considered all lead to a loss of vitality, except F31Y, which is neutral.

Table SM4: MMGBLK analysis of TMP binding

		Short minimization					Long minimization				
position		Total	vdW	Coul.	GB	LK	Total	vdW	Coul.	GB	LK
	F	7.9	6.6	-0.7	0.6	1.4	4.5	3.5	-0.6	0.4	1.2
28L	Y	4.2	2.3	-0.7	0.6	2.0	1.3	-0.2	-0.3	0.2	1.6
	W	3.2	0.4	1.6	-1.2	2.4	1.6	-0.6	1.1	-1.0	2.1
	M	19.5	21.2	1.0	-1.3	-1.4	1.9	2.7	2.7	-1.9	-1.6
	K	16.3	14.6	9.2	-6.6	-0.9	2.9	1.8	8.7	-6.8	-0.8
31F	E	6.8	7.9	-9.7	9.0	-0.4	0.9	2.4	-9.0	8.2	-0.7
	Y	3.7	3.1	1.0	-1.0	0.6	-0.3	-0.5	0.4	-0.6	0.4
	H	6.8	4.0	11.1	-8.7	0.4	1.7	0.0	9.9	-8.3	0.1

Free energies and free energy components (kcal/mol) for TMP binding.

2.7 Redesign of selected pairs

We report results for four pairs formed by positions 23, 28, 29, and 31. The top 15 variants are given for each pair in Table SM5.

Table SM5: Free energies and couplings (kcal/mol) from selected pair designs

Seq.	ΔG_{Vit}	$\Delta G^{DHF}_{\text{Bind}}$	$\Delta G^{TMP}_{\text{Bind}}$	$\Delta G^{Apo}_{\text{Fold}}$	Seq.	ΔG_{Vit}	$\Delta G^{DHF}_{\text{Bind}}$	$\Delta G^{TMP}_{\text{Bind}}$	$\Delta G^{Apo}_{\text{Fold}}$
		23N 28L					28L 29A		
IF	-9.4 (1)	1.4 (-1)	10.8 (-2)	0.7 (0)	HA	-1.2 (1)	1.0 (0)	2.2 (-1)	1.8 (0)
VF	-8.3 (1)	1.4 (-1)	9.8 (-2)	0.8 (0)	KK	-1.0 (1)	0.2 (0)	1.3 (-1)	2.4 (3)
RF	-8.3 (1)	1.1 (0)	9.4 (-1)	0.4 (0)	LK	-1.0 (1)	-0.4 (0)	0.6 (-1)	0.8 (0)
KF	-8.3 (1)	1.0 (0)	9.3 (-1)	0.7 (0)	LH	-0.9 (0)	-0.5 (0)	0.4 (-1)	1.4 (0)
HF	-8.2 (1)	1.1 (0)	9.3 (-1)	0.3 (0)	KT	-0.8 (1)	0.7 (0)	1.5 (-1)	1.7 (0)
MF	-7.5 (1)	1.4 (-1)	8.9 (-2)	1.1 (0)	LT	-0.8 (0)	0.1 (0)	0.9 (0)	0.3 (0)
LF	-7.3 (2)	1.4 (-1)	8.7 (-3)	0.7 (0)	TK	-0.8 (1)	0.7 (0)	1.5 (-1)	1.6 (0)
IH	-4.2 (-1)	0.8 (0)	5.0 (0)	0.9 (1)	LS	-0.6 (0)	0.1 (0)	0.6 (0)	0.9 (0)
VH	-3.0 (0)	0.8 (0)	3.9 (-1)	1.0 (1)	TT	-0.6 (0)	1.2 (0)	1.8 (-1)	1.1 (0)
RH	-3.0 (1)	0.6 (1)	3.5 (0)	1.0 (4)	LF	-0.3 (0)	0.1 (0)	0.5 (-1)	1.2 (0)
HH	-2.9 (0)	0.5 (1)	3.5 (0)	1.0 (6)	LY	-0.3 (0)	0.1 (0)	0.4 (0)	1.1 (0)
IK	-2.9 (0)	0.6 (1)	3.5 (0)	0.4 (0)	KR	-0.2 (0)	0.1 (0)	0.3 (0)	2.5 (3)
IL	-2.9 (0)	-0.1 (0)	2.8 (0)	-1.0 (0)	LW	-0.2 (0)	0.2 (0)	0.4 (0)	1.2 (0)
KH	-2.9 (2)	0.5 (1)	3.4 (-1)	1.4 (5)	LQ	-0.2 (0)	0.0 (0)	0.2 (-1)	1.2 (0)
QH	-2.7 (-1)	1.0 (0)	3.7 (1)	1.6 (0)	KL	-0.2 (1)	0.7 (0)	0.9 (-1)	1.8 (0)
		28L 31F					29A 31F		
FN	-8.5 (17)	-1.5 (1)	7.0 (-16)	3.4 (-1)	KQ	-4.9 (1)	-3.6 (1)	1.3 (0)	2.9 (0)
FQ	-8.3 (22)	-1.6 (2)	6.8 (-21)	3.6 (-2)	HQ	-4.8 (1)	-3.8 (0)	1.1 (0)	3.6 (0)
FC	-7.6 (27)	-2.6 (0)	5.0 (-27)	3.0 (-1)	TQ	-4.8 (0)	-3.1 (1)	1.6 (0)	2.5 (0)
FA	-7.5 (25)	-3.2 (-1)	4.2 (-26)	2.8 (-1)	KC	-4.7 (0)	-4.5 (1)	0.2 (1)	2.1 (-1)
FS	-7.3 (26)	-3.3 (0)	4.0 (-26)	4.0 (-1)	KL	-4.7 (0)	0.4 (0)	5.1 (0)	1.0 (0)
FT	-5.8 (22)	-0.4 (0)	5.4 (-22)	2.5 (-1)	HC	-4.6 (0)	-4.7 (1)	-0.1 (1)	2.8 (-1)
HQ	-5.2 (0)	-1.9 (5)	3.3 (4)	3.4 (-5)	KN	-4.6 (0)	-3.4 (1)	1.1 (0)	2.6 (0)
HA	-5.0 (-3)	-3.8 (0)	1.2 (3)	2.8 (-3)	HL	-4.5 (0)	0.3 (0)	4.8 (0)	1.7 (0)
HC	-4.9 (0)	-3.1 (1)	1.7 (1)	3.0 (-2)	SQ	-4.5 (1)	-3.1 (1)	1.3 (0)	3.0 (0)
HN	-4.8 (0)	-2.0 (1)	2.7 (1)	3.5 (-1)	TC	-4.5 (0)	-4.0 (1)	0.4 (1)	1.7 (-1)
HS	-4.6 (0)	-3.8 (1)	0.8 (1)	4.0 (-2)	KA	-4.5 (0)	-5.1 (0)	-0.6 (0)	2.0 (0)
LQ	-3.9 (1)	-3.2 (1)	0.6 (0)	2.1 (0)	HN	-4.5 (0)	-3.5 (1)	0.9 (0)	3.3 (0)
TQ	-3.7 (0)	-1.9 (3)	1.9 (3)	2.5 (-4)	KS	-4.4 (0)	-5.2 (1)	-0.8 (0)	3.1 (0)
KQ	-3.6 (4)	-2.3 (4)	1.3 (0)	3.0 (-4)	TL	-4.4 (0)	0.9 (0)	5.3 (0)	0.6 (0)
LL	-3.6 (0)	0.8 (0)	4.4 (0)	0.2 (0)	HA	-4.4 (0)	-5.2 (0)	-0.8 (0)	2.7 (-1)

Top 15 vitalities for 4 designed pairs, with DHF and TMP binding free energies and the apo-protein folding free energy. Coupling×10 in parentheses.

References

[1] Simonson, T. *The Proteus software for computational protein design.* https://proteus.polytechnique.fr, Ecole Polytechnique, Paris, 2019.

[2] Sawaya, M. R., and Kraut, J. Loop and subdomain movements in the mechanism of Escherichia coli dihydrofolate reductase: crystallographic evidence. *Biochemistry 36* (1997), 586–603.

[3] Jorgensen, W. L., Chandrasekar, J., Madura, J., Impey, R., and Klein, M. Comparison of simple potential functions for simulating liquid water. *J. Chem. Phys. 79* (1983), 926–935.

[4] Ryde, U. Molecular dynamics simulations of alcohol dehydrogenase with a four- or five-coordinate catalytic zinc ion. *Proteins 21* (1995), 40–56.

[5] Ryde, U. On the role of Glu68 in alcohol dehydrogenase. *Prot. Sci. 4* (1995), 1124–1132.

[6] Phillips, J. C., Braun, R., Wang, W., Gumbart, J., Tajkhorshid, E., Villa, E., Chipot, C., Skeel, R. D., Kale, L., and Schulten, K. Scalable molecular dynamics with NAMD. *J. Comput. Chem. 26* (2005), 1781–1802.

[7] Martyna, G. J., Tobias, D. J., and Klein, M. L. Constant pressure molecular dynamics algorithms. *J. Chem. Phys. 101* (1994), 4177–4189.

[8] Feller, S. E., Zhang, Y., Pastor, R. W., and Brooks, B. R. Constant pressure molecular dynamics simulation: the Langevin piston method. *J. Chem. Phys. 103* (1995), 4613–4622.

[9] Darden, T., York, D., and Pedersen, L. Particle mesh Ewald: an N log(N) method for Ewald sums in large systems. *J. Chem. Phys. 98* (1993), 10089–10092.

[10] Berman, H. M., Westbrook, J., Feng, Z., Gilliland, G., Bhat, T. N., Weissig, H., Shindyalov, I. N., and Bourne, P. E. The Protein Data Bank. *Nucl. Acids Res. 28* (2000), 235–242.

[11] Wan, Q., Bennett, B. C., Wilson, M. A., Kovalevsky, A., Langan, P., Howell, E. E., and Dealwis, C. Toward resolving the catalytic mechanism of dihydrofolate reductase using neutron and ultrahigh-resolution X-ray crystallography. *Proc. Natl. Acad. Sci. USA 111* (2014), 18225–18230.

[12] Tuffery, P., Etchebest, C., Hazout, S., and Lavery, R. A new approach to the rapid determination of protein side chain conformations. *J. Biomol. Struct. Dyn. 8* (1991), 1267–1289.

[13] Gaillard, T., and Simonson, T. Pairwise decomposition of an MMGBSA energy function for computational protein design. *J. Comput. Chem. 35* (2014), 1371–1387.

[14] Watson, M., Liu, J. W., and Ollis, D. Directed evolution of trimethoprim resistance in *Escherichia coli. FEBS Letters 274* (2007), 2661–2671.

[15] Tamer, Y. T., Gaszek, I. K., Abdizadeh, H., Batur, T. A., Reynolds, K. A., Atilgan, A. R., Atilgan, C., and Toprak, E. High-order epistasis in catalytic power of dihydrofolate reductase gives rise to a rugged fitness landscape in the presence of trimethoprim selection. *Molec. Biol. Evol. 36* (2019), 1533–1550.

[16] Manna, M. S., Tamer, Y. T., Gaszek, I., Poulides, N., Ahmed, A., Wang, X., Toprak, F. C., Woodard, D. R., Koh, A. Y., Williams, N. S., et al. A trimethoprim derivative impedes antibiotic resistance evolution. *Nat. Commun. 12* (2021), 1–10.

[17] Cammarata, M., Thyer, R., Lombardo, M., Anderson, A., Wright, D., Ellington, A., and Brodbelt, J. S. Characterization of trimethoprim resistant *e. coli* dihydrofolate reductase mutants by mass spectrometry and inhibition by propargyl-linked antifolates. *Chem. Sci. 8* (2017), 4062–4072.

[18] Abdizadeh, H., Tamer, Y. T., Acar, O., Toprak, E., Atilgana, A. R., and Atilgan, C. Increased substrate affinity in the *escherichia coli* L28R dihydrofolate reductase mutant causes trimethoprim resistance. *Phys. Chem. Chem. Phys. 19* (2017), 11416.

[19] Toprak, E., Veres, A., Michel, J. B., Chait, R., Hartl, D. L., and Kishony, R. Evolutionary paths to antibiotic resistance under dynamically sustained drug selection. *Nat. Gen. 44* (2012), 101–105.

[20] Huovinen, P. Trimethoprim resistance. *Antimicrob. Agents Chemother. 31* (1987), 1451–1456.

[21] Flensburg, J., and Skøld, O. Massive overproduction of dihydrofolate reductase in bacteria as a response to the use of trimethoprim. *Eur. J. Biochem. 162* (1987), 473–476.

[22] Michael, E., Polydorides, S., Simonson, T., and Archontis, G. Simple models for nonpolar solvation: parametrization and testing. *J. Comput. Chem. 38* (2017), 2509–2519.
